# FcγRIIb Inhibits Allergic Lung Inflammation in a Murine Model of Allergic Asthma

**DOI:** 10.1371/journal.pone.0009337

**Published:** 2010-02-22

**Authors:** Nilesh Dharajiya, Swapnil V. Vaidya, Hiroki Murai, Victor Cardenas, Alexander Kurosky, Istvan Boldogh, Sanjiv A. Sur

**Affiliations:** 1 National Heart, Lung, and Blood Institute (NHLBI) Proteomics Center, Department of Biochemistry and Molecular Biology, University of Texas Medical Branch, Galveston, Texas, United States of America; 2 Department of Internal Medicine, University of Texas Medical Branch, Galveston, Texas, United States of America; 3 Department of Biochemistry and Molecular Biology, University of Texas Medical Branch, Galveston, Texas, United States of America; 4 Department of Microbiology, University of Texas Medical Branch, Galveston, Texas, United States of America; New York University, United States of America

## Abstract

Allergic asthma is characterized by airway eosinophilia, increased mucin production and allergen-specific IgE. Fc gamma receptor IIb (FcγRIIb), an inhibitory IgG receptor, has recently emerged as a negative regulator of allergic diseases like anaphylaxis and allergic rhinitis. However, no studies to date have evaluated its role in allergic asthma. Our main objective was to study the role of FcγRIIb in allergic lung inflammation. We used a murine model of allergic airway inflammation. Inflammation was quantified by BAL inflammatory cells and airway mucin production. FcγRIIb expression was measured by qPCR and flow cytometry and the cytokines were quantified by ELISA. Compared to wild type animals, FcγRIIb deficient mice mount a vigorous allergic lung inflammation characterized by increased bronchoalveolar lavage fluid cellularity, eosinophilia and mucin content upon ragweed extract (RWE) challenge. RWE challenge in sensitized mice upregulated FcγRIIb in the lungs. Disruption of IFN-γ gene abrogated this upregulation. Treatment of naïve mice with the Th1-inducing agent CpG DNA increased FcγRIIb expression in the lungs. Furthermore, treatment of sensitized mice with CpG DNA prior to RWE challenge induced greater upregulation of FcγRIIb than RWE challenge alone. These observations indicated that RWE challenge upregulated FcγRIIb in the lungs by IFN-γ- and Th1-dependent mechanisms. RWE challenge upregulated FcγRIIb on pulmonary CD14+/MHC II+ mononuclear cells and CD11c+ cells. FcγRIIb deficient mice also exhibited an exaggerated RWE-specific IgE response upon sensitization when compared to wild type mice. We propose that FcγRIIb physiologically regulates allergic airway inflammation by two mechanisms: 1) allergen challenge mediates upregulation of FcγRIIb on pulmonary CD14+/MHC II+ mononuclear cells and CD11c+ cells by an IFN-γ dependent mechanism; and 2) by attenuating the allergen specific IgE response during sensitization. Thus, stimulating FcγRIIb may be a therapeutic strategy in allergic airway disorders.

## Introduction

Allergic asthma is an airway inflammatory disease that is characterized by bronchial hyper-responsiveness, airway eosinophilia, goblet cell hyperplasia and production of allergen specific IgE. Cross-linking of the high affinity IgE receptor (FcεRI) on mast cells by IgE, in the presence of allergen activates Btk, PLC-gamma and PI3K [1]–[4]. This ultimately leads to production and release of pro-inflammatory substances like histamine, leukotrienes and cytokines that promote allergic inflammation. In addition, cytokines produced by allergen specific Th2 cells such as IL4, IL5, IL9, IL13 and IL25 also promote allergen-specific IgE production and allergic airway inflammation [5]–[14].

There is considerable amount of data on pro-inflammatory mediators that contribute to the development of allergic inflammation. However, relatively little is known about negative regulatory mechanisms that attenuate allergic inflammation. FcγRIIb is an inhibitory low affinity IgG receptor expressed on many inflammatory cells, including monocytes, macrophages, dendritic cells, B cells, mast cells and basophils[15]. It negatively regulates innate and adaptive immune responses, and has been shown to inhibit activation of mast cells, basophils, B cells and T cells [16]–[19]. It is composed of two Ig-like extra-cellular domains that bind the Fc region of IgG, one trans-membrane domain and an intra-cytoplasmic tail with an immuno-receptor phospho-tyrosine based inhibitory (ITIM) motif [15], [20]. Activation of FcγRIIb leads to recruitment of phosphatases to the ITIM motif that inhibits signal transduction from other activating receptors[21]. This block in the signaling cascade is the main reason for its potent inhibitory effects on BCR-mediated B-cell activation, TCR-mediated T-cell activation and FceRI-mediated mast cell activation [17], [22]–[24]. This inhibitory role is evident from studies of FcγRIIb deficient mice that are more susceptible to auto-immune diseases and IgE mediated anaphylaxis [25]–[32]. Only a few studies have shown a regulatory role of this receptor in animal models of allergic diseases. One study showed that IgG antibodies can prevent IgE mediated anaphylaxis *in vivo* through both antigen interception and FcγRIIb cross-linking [33]. Another study demonstrated a regulatory role of FcγRIIb in a murine model of allergic rhinitis[34]. However, the role of this receptor in allergic lung inflammation has not been elucidated.

We recently showed in a gene micro-array analysis (GEO accession number GSE18083) that allergen challenge upregulated 352 genes in the lungs four hours after the challenge [35]. Careful review of that list revealed FcγRIIb as one of the genes upregulated. Based on this observation, we hypothesized that FcγRIIb may play a regulatory role in allergic airway inflammation. Here we show that mice lacking FcγRIIb have exaggerated allergic airway inflammation, suggesting its negative regulatory role in asthma. We further show that allergen challenge upregulates FcγRIIb in the lungs in an IFN-γ dependent mechanism. Our results indicate that FcγRIIb upregulation physiologically reduces allergic airway inflammation.

## Materials and Methods

### Ethics Statement

All animal experiments were approved by the Institutional Animal Care and Use Committee of the University of Texas Medical Branch at Galveston.

### Mice

Female BALB/c mice, 6–8 wk old, were purchased from the Harlan Laboratories (Indianapolis, IN). BALB/c IFN-γ KO, C57Bl6 FcγRIIb knock-out (KO) and C57Bl6 WT mice were purchased from Jackson laboratories (Bar Harbor, Maine). BALB/c FcγRIIb knock-out (KO) mice were purchased from Taconics (Albany, NY). All mice were maintained in a specific pathogen-free environment throughout the experiment.

### Model of Allergic Sensitization and Challenge

WT Balb/c, IFN-γ KO or FcγRIIb KO mice were sensitized by two intraperitoneal (i.p.) injections of endotoxin-free RWE (150 µg) and alum on days 0 and 4. On day 11, allergen challenge was performed by intranasal (i.n.) instillation of RWE (200 µg) in anesthetized mice. Mice were sacrificed at various time points as indicated after the challenge and bronchoalveolar lavage (BAL) fluid, blood, lung and spleen specimens were collected. Mice sensitized but not challenged served as the zero time point. For the Th1/CpG experiments, 35 µg CpG or GpC oligonucleotides were administered intranasally in 50 µl of sterile PBS [36].

### Ragweed Extract

We have previously shown that endotoxin contamination alters the inflammatory cell recruitment following allergen challenge [37]. To avoid this problem endotoxin-free ragweed (lot XP56-D10-1320) was purchased from Greer Laboratories (Lenoir, NC).

### Measurement of Allergic Airway Inflammation

For BAL fluid analyses, mice were anesthetized with an i.p. injection of ketamine and xylazine, tracheotomy performed and the trachea was cannulated. BAL of both lungs was performed twice with 0.7 ml of sterile PBS (pH 7.3) through the tracheal cannula with a syringe. Total cell counts were performed on BAL samples and differential cell counts were done on cytocentrifuge preparations (Cytospin 3; Thermo Shandon) stained with Wright-Giemsa, counting 200 cells from each animal. Mucin was quantified using mucin-binding lectin Jacalin (Calbiochem, La jolla, CA) as described previously [38]. Aliquots of BAL fluid were diluted 1∶100, 1∶1000 and 1∶10000, added in triplicate to individual wells of microtiter ELISA plates and incubated for 2 h at room temperature. Plates were washed and blocked with 5% BSA and 0.02% biotinylated jacalin was added. After 1 h incubation at room temperature, plates were washed extensively, then developed with alkaline phosphatase-conjugated avidin (Sigma) and nitrophenylphosphate (Sigma) and quantified by comparison with a mucin (Sigma) standard curve. The morphometric method we described previously was used to quantify mucin in lung epithelium[39]. Briefly, coronal sections of the 4% paraformaldehyde-fixed lungs were stained with PAS stain. Morphometirc analysis was done using Metamorph™ software (Version 5, Universal Imaging, Downingtown, Pennsylvania). Several images from five different levels per lung (three animals per group) were obtained and reassembled using the montage stage stitching algorithm of the Metamorph™ software. The integrated morphometric analysis function was used to transform total pixel area of the signal to µm^2^ per mm of peribronchial diameter.

### Measurement of Enhanced Pause Index (PENH Index)

PENH was assessed by a method previously described [40] using a dual chamber whole body plethysmograph obtained from Buxco (Troy, NY). Mice were exposed for 3 min to nebulized PBS and subsequently to 37.5 mg/ml nebulized methacholine (Sigma Chemicals) in PBS using the AeroSonic ultrasonic nebulizer. After each nebulization, recordings were taken for 4 min. The PENH values measured during each 4 min sequence were averaged and expressed as the percentage of baseline PENH values after PBS exposure.

### Quantification of Serum RWE-Specific IgE

Serum was collected from RWE-sensitized WT and FcγRIIb KO mice 4 h after challenge with PBS (WT PBS and KO PBS) or RWE (WT RWE and KO RWE). RWE-specific IgE was quantified using standard sandwich ELISA technique and comparison with an IgE standard curve as described previously[41].

### Antigen Recall Assay

Splenocytes were obtained from sensitized WT and FcγRIIb KO mice after crushing the spleens and making single cell suspensions. These were incubated with or without RWE for 3 d and Th2 cytokines (IL-4, IL-5, IL-9 and IL-13) were quantified in the supernatants using standard ELISA techniques as described previously [36], [41], [42].

### Quantitative RT-PCR

Balb/c mice sensitized with RWE were challenged with either RWE or PBS. Mice were sacrificed and lungs collected at 1, 4, 24, 72 and 240 h post-challenge. RNA was extracted and quantitative PCR analyses were performed using the SYBR green real time PCR kit (Applied Biosystems) as described previously [35], [43]. Transcript copy numbers for FcγRIIb and beta-actin were quantified by comparing to a standard curve generated from serial log-dilutions of the target DNA [44], [45]. FcγRIIb signal was normalized to beta-actin. Table 1 shows the primers used.

**Table 1:** Primers used for quantitative PCR analyses.

Gene	Forwad Primer	Reverse Primer
β-actin	ACACCTTCTACAATGAGCTG	GGATCTTCATGAGGTAGTCC
FcγRIIb	ATCTTGCTGCTGGGACTCAT	TGACTGTGGCCTTAAACGTG

### Flow Cytometry

Single cell suspensions of lung and spleen were analyzed by flow cytometry [46]. Cells were washed 3X with PBS and resuspended in FACS staining media containing 0.5% FBS in PBS. To study expression of FcγRIIb on dendritic cells, 1×10^6^ cells were incubated with anti-CD11c PE (Pharmingen, Clone HL3) and anti-CD16/CD32-biotin (Pharmingen, Clone 2.4G2) for 30 min on ice protected from light. After three washes, cells were incubated with Streptavidin Cy-chrome (Pharmingen, #554062). Species and isotype matched antibodies were used as controls. FACS analysis was performed using analytical Flow cytometer (FACS Scan, Beckton Dickinson) with CellQuest software (San jose, CA). Further analyses were performed using FlowJo software (Tree Star Inc., Ashland, OR). Similarly, FcγRIIb expression on macrophages (anti-CD14; Clone rmC5-3, Pharmingen and anti-MHC class II-FITC; Miltenyi biotech, #130-081-601) and B cells (anti-B220, Clone RA3-6B2, Pharmingen) was studied.

### Statistical Methods

There were 4–6 animals in each group and results are representative of at least two independent experiments. Statistical significance between groups was determined using Student's T test.

## Results

### Disruption of the FcγRIIb Gene Augments Allergic Airway Inflammation

**Figure 1:**
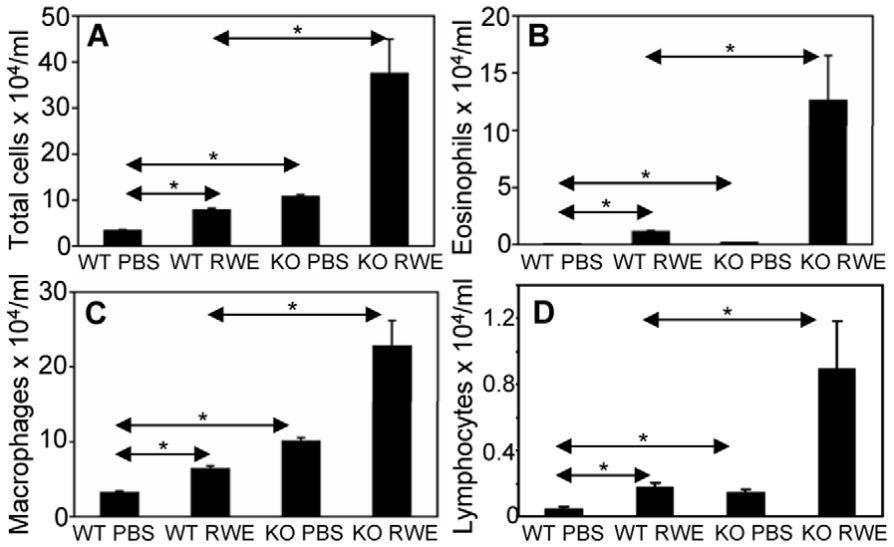
Role of FcγRIIb in allergic airway inflammation. (A, B, C and D) Total inflammatory cells (A), eosinophils (B), macrophages (C) and lymphocytes (D) were quantified in BAL of C57Bl6 RWE-sensitized WT and FcγRIIb KO mice challenged with either PBS (WT PBS and KO PBS) or RWE (WT RWE and KO RWE). *, p<0.05.

**Figure 2:**
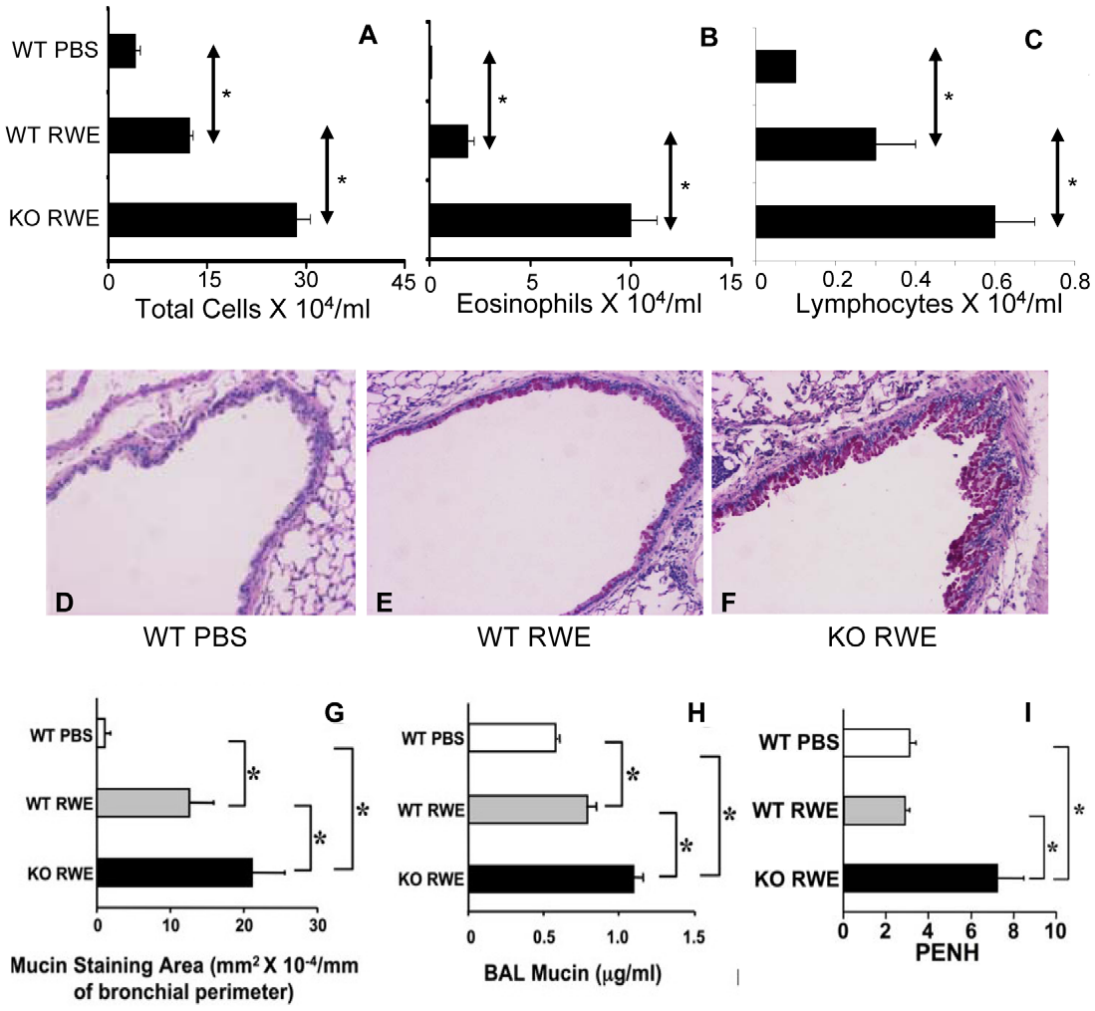
Role of FcγRIIb in allergic airway inflammation. (A, B and C) Total inflammatory cells (A), eosinophils (B) and lymphocytes (C) were quantified in BAL of Balb/c RWE-sensitized WT and FcγRIIb KO mice challenged with either PBS (WT PBS) or RWE (WT RWE and KO RWE). (D, E and F) Lung sections were obtained from RWE-sensitized WT and FcγRIIb KO mice challenged with either PBS (WT PBS) or RWE (WT RWE & KO RWE). These sections were stained with PAS to identify mucin containing cells. (G) Mucin containing cells in the lung sections were analyzed by morphometric analyses of PAS staining area. (H) Mucin was quantified in BAL samples by ELISA using biotinylated mucin binding lectin. (I) WT and FcγRIIb KO mice were sensitized with RWE and challenged with either PBS (WT PBS) or RWE (WT & KO RWE). PENH was measured by Buxco whole body plethysmography. *, p<0.05.

We assessed the biological role of FcγRIIb in a murine model of allergic asthma. C57Bl6 wild type (WT) and C57Bl6 FcγRIIb knock-out (KO) mice were sensitized and then challenged with RWE. RWE challenge in WT mice recruited 3-fold more total inflammatory cells, 10-fold more eosinophils, 2-fold more lymphocytes and macrophages (**Figure 1A, 1B, 1C and 1D**). Disrupting the FcγRIIb gene further increased total inflammatory cells (5-fold), eosinophils (12-fold), lymphocytes (5-fold) and macrophages (3.6-fold) in the BAL (**Figure 1A, 1B, 1C and 1D**). To determine the reproducibility of this result in a different strain of mouse, we repeated this experiment in Balb/c mice. RWE challenge in WT Balb/c mice recruited 3-fold more total inflammatory cells, 32-fold more eosinophils and 3-fold more lymphocytes in BAL as compared to PBS challenge (**Figure 2A, 2B and 2C**) at 72 h post-challenge. Similar to our observations in C57Bl6 mice, RWE challenge in Balb/c FcγRIIb KO mice further increased total cells (2.3-fold increase), eosinophils (5.2-fold increase) and lymphocytes (2-fold increase) in BAL fluid as compared to WT mice (**Figure 2A, 2B and 2C**). RWE challenge in WT mice increased mucin-containing cells in the airway (**Figures 2D, 2E and 2G**) and mucin levels in BAL fluid (**Figure 2H**). RWE challenge in mice that lacked FcγRIIb further increased mucin-containing cells in the airway (**Figures 2E, 2F and 2G**) and mucin levels in BAL fluid (**Figure 2H**) as compared to WT mice. RWE challenge in mice that lacked FcγRIIb induced greater increase in enhanced pause (PENH) index as compared to wild type mice (**Figure 2I**).

### RWE Challenge Upregulates FcγRIIb in the Lungs by an IFN-γ-Dependent Mechanism

**Figure 3:**
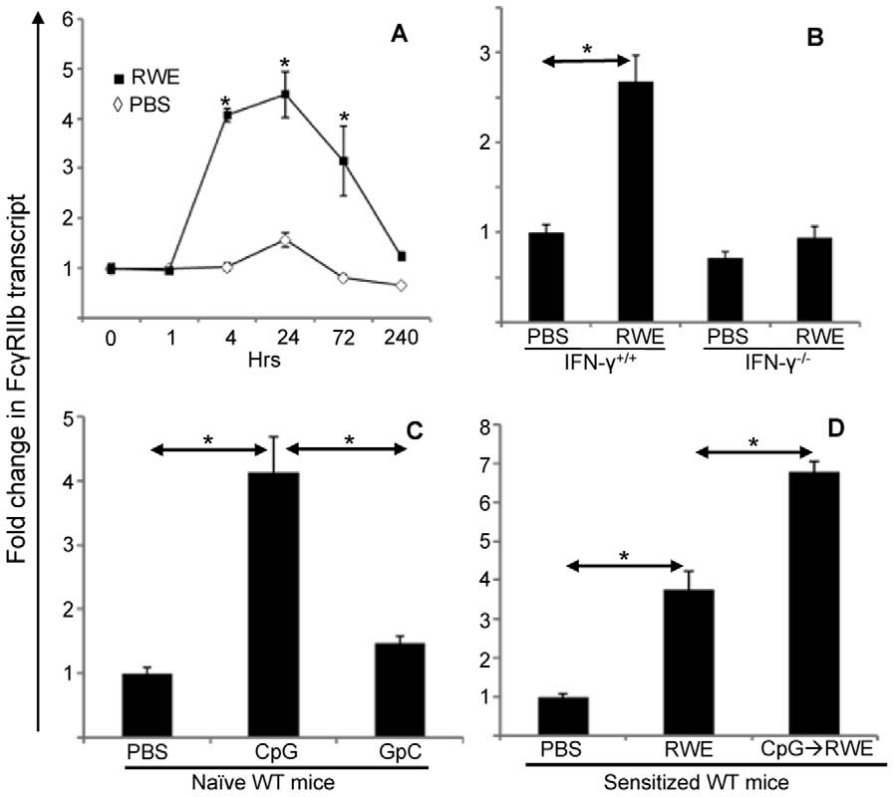
Expression of FcγRIIb in the lungs after RWE challenge. (A) Balb/c mice sensitized with RWE and challenged with either RWE (filled squares) or PBS (open diamond). Mice were sacrificed 1, 4, 24, 72 and 240 h after challenge, lungs were collected and RNA was extracted. Quantitative PCR (qPCR) analysis for FcγRIIb was performed on these RNA samples using SYBR green Real time PCR kit (Applied biosystems). (B) Wild-type and INF-γ deficient BALB/c mice were sensitized with RWE, and challenged with PBS or RWE. 4 h later, the lungs were collected and qPCR for FcγRIIb was performed. (C) Naïve wild-type mice were challenged with PBS, CpG DNA or GpC DNA. 4 hours post-challenge lungs were collected and FcγRIIb expression was quantified by qPCR. (D) Wild-type BALB/c mice were sensitized with RWE. The mice were pre-treated with PBS (PBS challenge or RWE challenge group) or 35 µg CpG oligonucleotide intranasally (CpG → RWE) 48 h prior to RWE challenge. 4 h post-challenge, lungs were collected and qPCR for FcγRIIb was performed. * = p<0.05.

Since allergen challenge recruits inflammatory cells that express FcγRIIb to the lungs, and lack of FcγRIIb further increases this inflammation, we hypothesized that allergen challenge upregulates FcγRIIb on pulmonary inflammatory cells. Quantitative PCR of lung mRNA confirmed that RWE challenge upregulated FcγRIIb as early as 4 hours post-RWE challenge, and gene expression peaked at 24 h (**Figure 3A**). This upregulation was sustained till 10 d after challenge (**Figure 3A**). Prior studies have shown that IFN-γ and Th1 response can inhibit allergic inflammation [39], [47]–[50]. Since our studies suggested that FcγRIIb inhibited allergic airway inflammation, we sought to determine whether its upregulation was Th1 or IFN-γ dependent. RWE challenge upregulated FcγRIIb in wild type mice but not in IFN-γ KO mice (**Figure 3B**). IFN-γ KO mice also exhibited greater allergic airway inflammation when compared to WT mice (data not shown). Treatment of naïve wild type mice with the Th1-inducing CpG DNA significantly upregulated FcγRIIb; however, GpC control DNA (which does not induce IFN-γ) failed to do so (**Figure 3C**). Furthermore, intra-nasal administration of CpG DNA, but not GpC DNA, 48 h prior to RWE challenge in wild type mice enhanced RWE-induced FcγRIIb upregulation (**Figure 3D**). These findings indicated that RWE-challenge upregulated FcγRIIb by an IFN-γ and Th1-dependent mechanism.

### RWE Challenge Upregulates FcγRIIb in CD14+ MHC II+ Mononuclear Cells and CD11c+ Cells in the Lungs

**Figure 4:**
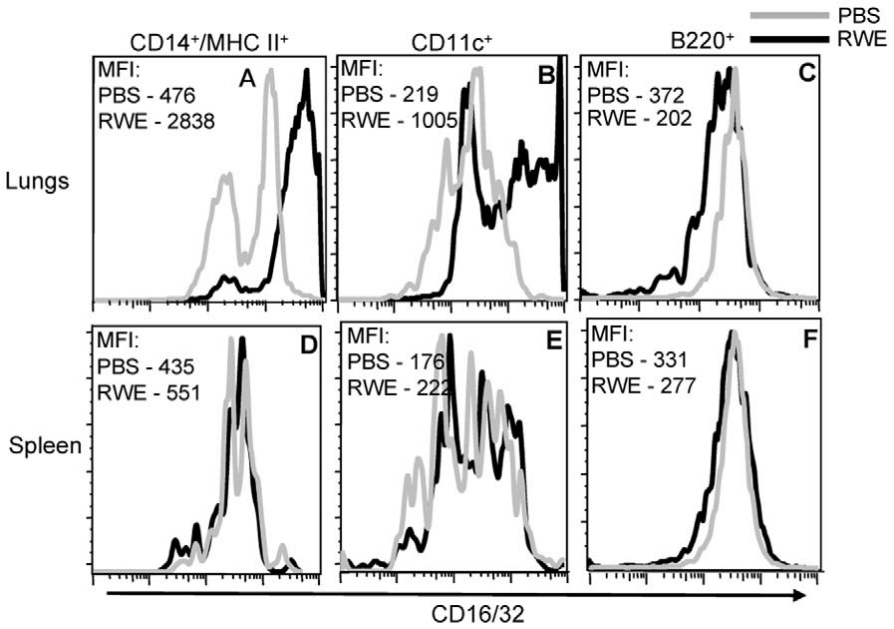
Identification of cells in the lungs that upregulate FcγRIIb after RWE challenge. Single cell lung and spleen suspensions were prepared from RWE-sensitized BALB/c mice that were challenged with PBS or RWE. A multi-color FACS analysis for FcγRIIb and cell specific markers (CD14/MHC II for macrophages, CD11c for dendritic cells and B220 for B cells) was performed on these cells. FcγRIIb expression is shown for PBS challenged (grey histogram) and RWE challenged (black histogram) mice. FcγRIIb expression is increased on CD14+/MHC II+ and CD11c+ gated cells. Data from one representative animal in each group. MFI, Mean fluorescence intensity.

We verified the upregulation of FcγRIIb in the lungs by flow cytometry measurements of single cell suspensions of whole lungs. RWE challenge upregulated FcγRIIb on pulmonary CD14+/MHC II+ cells (**Figure 4A**) and on CD11c+ cells (**Figure 4B**), but not on B220+ cells (**Figure 4C**). Furthermore, intrapulmonary RWE challenge failed to upregulate FcγRIIb expression on these cells in the spleen (**Figures 4D, 4E and 4F**). This suggested that RWE challenge upregulated FcγRIIb expression on CD14+/MHC II+ and CD11c+ cells in the challenged organ (lungs) with no detectable systemic upregulation.

### Disruption of the FcγRIIb Gene Augments Serum RWE-Specific IgE Levels after Antigen Sensitization, but Does Not Affect Th2 Cytokine Production in Antigen Recall Assay

**Figure 5:**
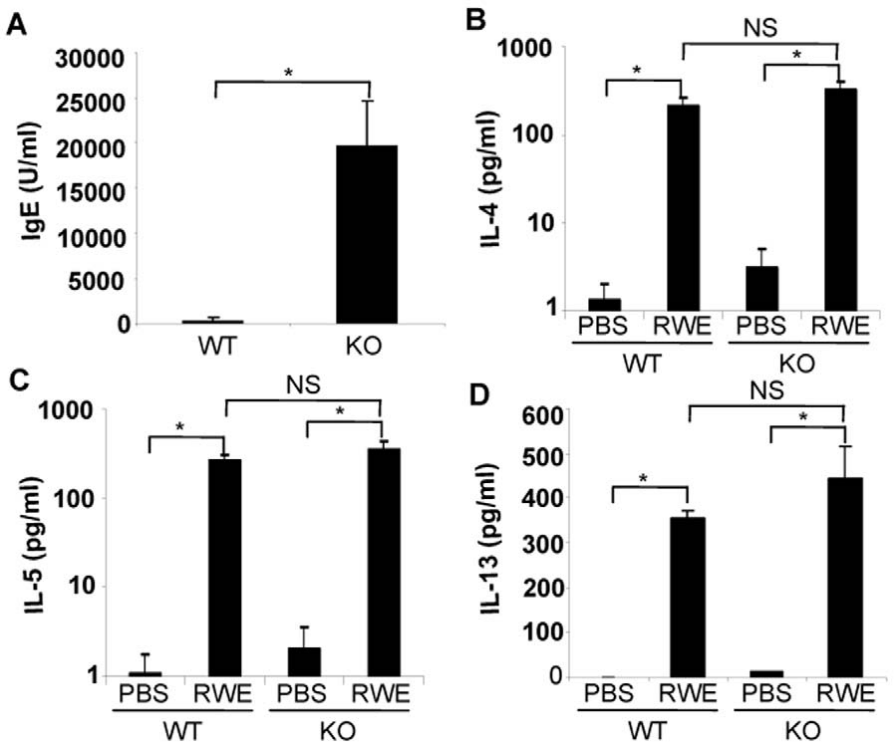
Role of FcγRIIb on serum IgE levels and antigen-induced Th2 cytokine production. (A) RWE-specific IgE levels in serum were quantified in sensitized WT and FcγRIIb KO mice. (B, C and D) Splenocytes from sensitized wild-type and FcγRIIb KO mice were cultured with PBS or RWE for four days, and the cell supernatants were analyzed for IL-4, IL-5 and IL-13 levels by ELISA. *, P<0.05; NS, not significant.

Building on the observation that FcγRIIb regulated RWE challenge induced allergic lung inflammation, we examined its role in the sensitization process and antigen-driven Th2 cytokine production. As shown in **Figure 5A**, sensitized FcγRIIb KO mice had significantly higher RWE-specific IgE levels when compared to WT mice. We hypothesized that this enhanced IgE response in FcγRIIb KO mice was due to an exaggerated Th2 response. To test this hypothesis we performed an antigen recall assay using splenocytes from sensitized WT and FcγRIIb KO mice. Importantly, IL-4, IL-5 and IL-13 production in response to RWE was similar in WT and FcγRIIb KO mice (**Figures 5B, 5C and 5D**). Thus, disruption of FcγRIIb increased antigen-specific IgE levels *in vivo* without increasing antigen-induced Th2 cytokine production.

## Discussion

FcγRIIb is an inhibitory IgG receptor that can prevent BCR-, TCR- and FcεRI-mediated activation of B-, T- and mast cells by recruitment of SHIP to its ITIM motif [17], [51]–[54]. Multiple studies have looked at the role of FcγRIIb in down regulating specific allergic inflammatory cells *in vitro*. However, only a few studies have demonstrated its regulatory role in animal models of allergic disease. One study showed that disruption of FcγRIIb increased nasal eosinophilia in mice sensitized and challenged with Schistosoma egg antigen (SEA)[34]. Another study suggested a role of upregulated FcγRIIb in the inhibition of anaphylaxis[55]. In this study we demonstrated the role of FcγRIIb in regulating allergen-induced eosinophilic inflammation in the lungs. We further showed for the first time that allergen challenge upregulated FcγRIIb in the lungs.

The genes that regulate FcγRIIb expression in the lungs have not been described. Here we demonstrate that IFNγ plays a critical role in mediating allergen-induced FcγRIIb upregulation. We recently showed that IFNγ plays an important role in upregulating Th1-associated genes such as p47 and p65 GTPases, Socs1, Cxcl9 and Cxcl10 after allergen challenge [35]. Our observations in the current manuscript indicate that FcγRIIb is another allergen-induced IFNγ-dependent, CpG DNA inducible gene. Other reports have demonstrated upregulation of FcγRIIb on naïve human blood-derived monocytes and dendritic cells by IL-4[56], [57]. This apparent disparity between mice and humans in regulation of FcγRIIb by Th1 and Th2 cytokines could be due to tissue-specific differences in the regulation of FcγRIIb, or may reflect divergence in regulation of the gene in the two species.

RWE challenge upregulated FcγRIIb on pulmonary CD14+/MHC II+ macrophages in this study. Alveolar macrophages have been shown to play a regulatory role in airway inflammation. Monocytes/macrophages account for a large number of cells in the airway in quiescent asthma. Removal of macrophages from the airways of patients with asthma by BAL enhances eosinophilic inflammation[58]. There could be several mechanisms by which alveolar macrophages contribute to this regulatory function. Macrophages express functional FcεRI and cross-linking leads to activation and secretion of pro-inflammatory cytokines[59], [60]. It is possible that the balance of expression of FcγRIIb and FcεRI by alveolar macrophages determines a pro-inflammatory versus anti-inflammatory role of these cells. In the present study, RWE challenge also upregulated CXCL9 and CXCL10 (data not shown), which are Th1-associated chemokines that have been shown to inhibit allergic airway inflammation[61], [62]. It is possible that airway monocytes secrete these anti-inflammatory cytokines upon FcγRIIb ligation, and mediate attenuation of allergic inflammation. Another possibility is that RWE challenge induces the anti-inflammatory PGE2 by macrophages in an FcγRIIb dependent fashion[63].

Allergen challenge also upregulated FcγRIIb on pulmonary CD11c+ cells, most likely dendritic cells. Myeloid dendritic cells have been shown to regulate allergic airway inflammation by inducing a Th2 immune response[64], [65]. FcγRIIb on DCs can potentially inhibit the induction of the Th2 cytokine response. However in the present study, the antigen recall assay failed to show an increase in IL-4 and IL-5 production in FcγRIIb knockout mice. These observations suggested that FcγRIIb does not affect the antigen presenting and Th2 skewing properties of DCs.

A previous study showed a critical role of Fc receptor gamma chain in the sensitization phase of allergic airway inflammation [66]. In the present study, absence of FcγRIIb increased levels of allergen specific IgE after sensitization. This indicated that FcγRIIb can specifically attenuate IgE humoral responses, suggesting its specific regulatory role in allergic lung inflammation. IgE production by the differentiating B cell requires class switch recombination (CSR) to Cε that is CD40 and IL-4 dependent[67], [68]. FcγRIIb deficient splenocytes made similar amount of IL-4 as wild type splenocytes in allergen recall assay. Thus, T cell-secreted IL-4 might not be involved in the FcγRIIb-mediated suppression of Cε class switch. One possibility is that FcγRIIb suppresses CD40L expression on T cells thus reducing the stimulus for IgE class switch. Another mechanism might involve regulation of IgE production by DCs. CSR in B cells is regulated by the expression of BAFF (BLyS) and APRIL on DCs[69]–[71]. One report showed inhibition of B cell IgE production by DCs via direct cell-cell interaction as well as by soluble factors including TGF-β and IFN-γ[72]. It is possible that FcγRIIb expression affects the ability of DCs to regulate IgE production by B cells. Yet another possibility is that the enhanced IgE response in FcγRIIb deficient mice is independent of the Th2 T cell response.

Upregulation of FcγRIIb on mast cells after exposure to allergen can lead to co-ligation of FcγRIIb and FcεRI by allergen and inhibit activation/degranulation of the mast cell. This concept was exploited in recent studies using two novel bio-engineered fusion proteins, one that consists of human Fc regions of IgG1 and IgE linked together and another a fusion protein made by linking an allergen to human IgG1 Fc region[73]. These proteins block pro-inflammatory mediator and cytokine release from allergic cells and prevent skin, lung and systemic allergic reactivity in a murine model[16], [73]–[77]. Our study demonstrates that FcγRIIb-dependent regulatory mechanism(s) control allergic airway inflammation, making this inhibitory receptor a physiologically relevant therapeutic target in allergic asthma. FcγRIIb appears to inhibit both allergic sensitization (possibly by attenuating the IgE response) as well as allergic inflammation from allergen exposure (possibly by upregulating FcγRIIb expression on inflammatory cells in the target organ). Stimulating the inhibitory FcγRIIb receptor is an elegant strategy because it is naturally upregulated by allergen exposure, and has the potential of controlling allergic inflammation by inhibiting multiple cells and mediators. In this manner it is likely to alter airway remodeling and disease progression.
